# Healthcare personnel in 2016–2019 prospective cohort infrequently got vaccinated, worked while ill, and frequently used antibiotics rather than antivirals against viral influenza illnesses

**DOI:** 10.1111/irv.13189

**Published:** 2023-09-07

**Authors:** Eduardo Azziz‐Baumgartner, Joan Neyra, Tat S. Yau, Giselle Soto, Daniel Owusu, Chao Zhang, Candice Romero, Young M. Yoo, Miriam Gonzales, Yeny Tinoco, María Silva, Eduar Bravo, Nancy Rojas Serrano, Eduardo Matos, Victor Chavez‐Perez, Juan Carlos Castro, Maria Esther Castillo, Rachael Porter, Cesar Munayco, Angel Rodriguez, Min Z. Levine, Michael Prouty, Mark G. Thompson, Carmen Sofia Arriola

**Affiliations:** ^1^ Influenza Division Centers for Disease Control and Prevention Atlanta Georgia USA; ^2^ US Naval Medical Research Unit No. 6 Bellavista Peru; ^3^ Vysnova Partners Inc Lima Peru; ^4^ Hospital Cayetano Heredia Lima Peru; ^5^ Instituto Nacional de Salud Lima Peru; ^6^ Hospital Nacional Arzobispo Loayza Lima Peru; ^7^ Hospital Nacional Dos de Mayo Lima Peru; ^8^ Hospital Nacional Daniel Alcides Carrión Lima Peru; ^9^ Instituto Nacional de Salud del Niño Lima Peru; ^10^ Medicine School from Universidad Peruana Cayetano Heredia Lima Peru; ^11^ Peruvian Centers for Disease and Control Lima Peru; ^12^ Health Emergencies Department, Pan American Health Organization (PAHO/WHO) Washington District of Columbia USA

**Keywords:** acute respiratory illness, healthcare personnel, influenza, influenza vaccine

## Abstract

**Background:**

Uncertainty about risk of illness and the value of influenza vaccines negatively affects vaccine uptake among persons targeted for influenza vaccination.

**Methods:**

During 2016–2019, we followed a cohort of healthcare personnel (HCP) targeted for free‐of‐charge influenza vaccination in five Lima hospitals to quantify risk of influenza, workplace presenteeism (coming to work despite illness), and absenteeism (taking time off from work because of illness). The HCP who developed acute respiratory illnesses (ARI) (≥1 of acute cough, runny nose, body aches, or feverishness) were tested for influenza using reverse‐transcription polymerase chain reaction (rt‐PCR).

**Findings:**

The cohort (2968 HCP) contributed 950,888 person‐days. Only 36 (6%) of 605 HCP who participated every year were vaccinated. The HCP had 5750 ARI and 147 rt‐PCR‐confirmed influenza illnesses. The weighted incidence of laboratory‐confirmed influenza was 10.0/100 person‐years; 37% used antibiotics, and 0.7% used antivirals to treat these illnesses. The HCP with laboratory‐confirmed influenza were present at work while ill for a cumulative 1187 hours.

**Interpretation:**

HCP were frequently ill and often worked rather than stayed at home while ill. Our findings suggest the need for continuing medical education about the risk of influenza and benefits of vaccination and stay‐at‐home‐while‐ill policies.

## 1 INTRODUCTION

In many countries, influenza vaccines and antivirals are infrequently used, partly because persons targeted for vaccination (e.g., healthcare personnel [HCP]) are unsure about their risk and the value of vaccines and antivirals to prevent severe influenza illness.^1^ This is especially true in middle‐income countries with recently established vaccine programs and where there is often little information about influenza. The World Health Organization (WHO) and Pan‐American Health Organization (PAHO)'s Technical Advisory Groups recommend vaccination of HCP against influenza^2^ to protect HCP from illnesses and prevent their workplace presenteeism (coming to work despite illness) and absenteeism (time taken off from work due to illness).^3^, ^4^ Vaccinating HCP against influenza has additional benefits, including preventing nosocomial influenza illnesses^3^ and more frequent HCP recommendation of influenza vaccines to their patients.^1^ Consistent with the WHO and PAHO recommendations, the government of Peru recommends and purchases influenza vaccines for their administration among HCP, free‐of‐charge, in government‐subsidized health clinics.^5^ Influenza vaccine uptake among HCP, however, is low.^6^ In a 2009–2010 mixed‐method study, for example, Peruvian physicians were less likely to get vaccinated than other target groups because they believed that: (1) they were not in contact with influenza patients; (2) influenza illness was mild; or (3) influenza was absent in their communities.^6^ Low vaccine uptake, because of uncertainty about the risk of influenza and the value of prevention, seems common,^7^ even in countries that mandate and provide influenza vaccines free‐of‐charge^8^ and is associated with suboptimal epidemic and pandemic controls.^9^

Despite HCP uncertainty, HCP may be at disproportionately high risk of influenza infections. In a meta‐analysis of publications dating from 1947 to 2020, HCP were 3.4 (95% CI, 1.2 to 5.7) times more likely to contract influenza infection when compared with nonmedical personnel in the community.^10^ Although occupational exposure may increase risk of infection among HCP, household exposure may be a significant factor in the increased risk of influenza among HCP.^11^ Multi‐year and site studies indicate that influenza is prevalent among Peruvian households during well‐defined epidemics, which typically occur May–September in Lima,^12^ but there is limited information about the risk of influenza and the uptake of influenza vaccines among Peruvian HCP.

To better understand the risk of illness and the utility of vaccines, we established a multi‐year cohort to quantify the incidence rate of influenza illnesses among HCP. Here, we quantify the risk of influenza illnesses, presenteeism, and absenteeism and assess demographic, household, and occupational factors associated with increased risk of laboratory‐confirmed influenza among HCP. HCP in Peru are entitled to 3 days of paid sick leave but require an Occupational Health Office evaluation and certification for absences of more than 3 days. The aim of this study was to investigate the incidence of ARI and specifically influenza in HCP across five hospitals in Lima, quantify their attendance or absence from work when ill with influenza, their medication use for influenza illness, and explore household and occupational risk factors for influenza illness. Such findings were anticipated to better establish the value of influenza prevention and treatment for HCP subpopulations in low‐ and middle‐income countries like Peru. As secondary objectives, we quantified the percent of unvaccinated HCP who, despite not having ARI, had a rise in influenza antibody titers from the pre‐ to post‐influenza season serology, suggestive of asymptomatic influenza infection or paucisymptomatic illness and the proportion who developed ARI because of other common respiratory viruses.

## 2 METHODS

We enrolled a stratified random sample of HCP working in five Lima hospitals for follow‐up twice a week to identify acute respiratory illnesses (ARI), obtain nasal swabs, and identify influenza through real time reverse transcription polymerase chain reaction (rt‐PCR).^13^ The cohort, Vacunas de Influenza Peru (VIP), comprised unvaccinated HCP aged ≥18 years prior to each influenza season from the Dos de Mayo National Hospital, Cayetano Heredia National Hospital, Daniel Alcides Carrión National Hospital, National Institute of Child Health, and Archbishop Loayza Hospital. These five hospitals are located in Lima, Peru and are level III specialty care national reference hospitals with 2500–3000 HCP, including clinical care personnel and ancillary staff. HCP at these hospitals were offered N95 mask fit test training and a variety of personal protective equipment to promote infection control (e.g., when conducting aerosolizing procedures).

To minimize selection bias, we sought to enroll ≥50 participants by sex, age group (i.e., 18–34, 35–49, and ≥50 years), and occupational (i.e., physicians, nurses/technicians, and assistants) strata. First, the study staff met with hospital administrators to determine which HCP belonged to which study strata. We generated random numbers in the spreadsheet with the HCP census and selected the first 50 within each of the nine age, sex, and occupation strata. Then, the staff invited this random selection of ≥50 participants from each stratum to participate in the VIP cohort. HCP were eligible to participate if they were aged ≥18 years; had hands‐on or face‐to‐face contact with patients within 1 m, as part of regular shifts; worked ≥30 hours per week; were employed for ≥1 year; planned to continue working at the hospital for ≥2 weeks; and owned a cellular or mobile phone to receive short‐message‐service (SMS) texting.

In an enrollment survey,^13^ participants self‐described their education, income, well‐being in the 24 hours prior to the survey (where higher numbers on a scale of 0%–100% indicate better wellness),^14^ socioeconomic status, tobacco and alcohol consumption, exercise habits, preexisting conditions, vaccinations, dwelling size, household members, role in the hospital, type and duration of patient contact, personal protective equipment use, days per week of physical exercise, age or pregnancy status of patients, procedures performed, self‐reported history of N95% fit testing or being given a respirator that fit the face, types of personal protective equipment HCP commonly wore when performing aerosolizing procedures, and other infection control practices. The same survey was administered in Peruvian Spanish at the beginning of each season among newly enrolled or reenrolled HCP at study hospitals. We calculated body mass index by dividing self‐reported weight in kilograms by height in meters squared.

Trained staff obtained pre‐ and post‐season sera from all participants and initiated active surveillance during the May–September influenza season.^13^ Sera were analyzed by hemagglutination inhibition assays against influenza viruses as described previously.^13^ The staff contacted the participants through SMS texts twice a week to assess whether they had developed ARI within the last 7 days. ARI was defined as ≥1 of acute cough, runny nose, body aches, or feverishness. A reminder SMS message was sent within 24 hours. If the HCP missed two consecutive SMS messages, the study staff would then call the non‐responders. The HCP were provided phone credits as a general incentive for participation in the study.

The HCP that reported ARI were given brief acute illness surveys that documented symptoms, illness duration and severity, workplace absenteeism^4^ or presenteeism, health seeking, treatment, and history of ill contacts. Presenteeism and absenteeism data were not collected in 2019 because of insufficient funds for these objectives. The HCP with ARI also provided self‐ or staff‐collected nasal swabs, which were transported to the US Naval Medical Research Unit No. 6 each day in cool boxes for rt‐PCR testing for influenza ribonucleic acid (RNA) through WHO recommended methods using reagents and protocols provided by the Centers for Disease Control and Prevention (CDC).^15^ After 7 days, the study staff texted the HCP every other day, up to three times, to assess whether they were still ill. Each year, a convenience sample of approximately 200 ARI, which tested negative for influenza RNA through rt‐PCR, were tested through FilmArray respiratory panel.^16^ This automated PCR platform is FDA‐approved and simultaneously detects 17 respiratory viruses and three bacterial targets.

### 2.1 Statistical analysis

To calculate rates of laboratory‐confirmed influenza illness, we divided the number of laboratory‐confirmed influenza by the person‐time HCP contributed to the cohort during the 2016–2019 influenza epidemic periods (i.e., the period when influenza was detectable among persons sampled through influenza surveillance in Peru). Among the HCP who reported ARI but did not provide swabs for influenza testing, we estimated the number of laboratory‐confirmed influenza illnesses by multiplying the ARI number by the proportion of persons who tested positive for influenza in Peru each epidemic week of the study.^17^ The study sampling weights were then applied to the influenza‐associated ARI rate after correcting for under‐ascertainment of laboratory‐confirmed influenza among HCP with ARI who did not provide a timely quality swab.^18^

ARIs were considered separate events if they started more than 14 days after the last day of reported symptoms from the previous ARI. We calculated the HCP person‐time as the difference between the first day the HCP were asked about the symptoms and the last day of follow‐up after each season, minus the weeks when the HCP were unreachable, the duration of ARI, and a 14‐day post‐ARI refractory period where we assumed that the HCP could not develop a new ARI. The risk of ARI and laboratory‐confirmed influenza was expressed as the number of ARIs and laboratory‐confirmed influenza illnesses per 100 person‐years (py) contributed to the cohort.

We used the univariate Poisson regression with person‐time as an offset term to explore demographic, household, and occupational factors associated with relative risk of ARI for all enrolled HCP, and we used the multivariable Poisson regression model to estimate the risk of ARI. Similarly, we used the multivariable Poisson regression to calculate rate ratios of influenza illness only for the HCP with laboratory‐confirmed results, after removing the HCP with ARI who did not provide timely quality swabs. Generalized estimating equations (GEEs) were used to account for the yearly reenrollment of participants during 2016–2019. In all adjusted models, we included age, sex, and occupation *a priori*, and any variables of interest (Table 1 and Table A1) that were significantly associated with ARI and/or laboratory‐confirmed influenza with a *p*‐value of ≤0.05 in the univariate analyses, controlling for the sampling weights.

**TABLE 1:** Demographic characteristics and respiratory illnesses among healthcare personnel—Vacunas de influenza Peru, 2016–2019.

Variable	Year of enrollment					*P*‐value^a^	
	2016	2017	2018	2019	Total	ARI	Influenza
Sample size	1116	2616	2167	1513	2968	‐	‐
Age at enrollment, median (IQR)	41 (33–52)	40 (32–51)	41 (33–51)	41 (33–52)	41 (32–51)	**0.050**	0.902
Body mass index, median (IQR)	25.9 (23.6–28.7)	26.0 (23.7–28.7)	26.2 (23.8–28.8)	26.2 (23.8–28.7)	26.0 (23.7–28.8)	0.613	0.841
Female (%)	803 (72)	1911 (73)	1580 (73)	1132 (75)	2138 (72)	**0.000**	0.483
Race^b^ (%)						0.505	0.993
Mixed	997 (89.7)	2335 (89.5)	1945 (89.8)	1343 (88.8)	2640 (89.3)	‐	‐
Quechua	53 (4.8)	130 (5.0)	105 (4.9)	80 (5.3)	153 (5.2)	‐	‐
Afro‐Peruvian	17 (1.5)	52 (2.0)	40 (1.9)	38 (2.5)	58 (2.1)	‐	‐
White or European	14 (1.3)	33 (1.3)	28 (1.3)	18 (1.2)	38 (1.3)	‐	‐
Asian	13 (1.2)	21 (0.8)	20 (0.9)	15 (1.0)	26 (0.9)	‐	‐
Amazonian	5 (0.4)	16 (0.6)	13 (0.6)	5 (0.3)	16 (0.5)	‐	‐
Aymara	7 (0.6)	11 (0.4)	6 (0.3)	9 (0.6)	13 (0.4)	‐	‐
Other	5 (0.5)	12 (0.5)	10 (0.5)	5 (0.3)	13 (0.4)	‐	‐
Household							
Married (%)	477 (43)	1011 (39)	839 (39)	593 (39)	1143 (38.5)	0.583	**0.037**
Children & adolescents aged ≤18 years	673 (60.4)	1605 (61.4)	1348 (62.2)	968 (64.0)	1837 (61.9)	0.933	0.116
Age of youngest person in household, median (IQR)	12 (5–24)	12 (5–24)	12 (4–24)	12 (4–23)	12 (4–24)	**0.010**	0.293
Children ≤5 years	268 (24.0)	630 (24.1)	531 (24.5)	391 (25.8)	737 (24.8)	0.533	0.408
Number of household members, median (IQR)	3 (2–4)	3 (2–4)	3 (2–4)	3 (2–4)	3 (2–4)	0.597	0.407
Persons per room, median (IQR)	1 (0.8–1.3)	1 (0.8–1.3)	1 (0.8–1.3)	1 (0.8–1.3)	1 (0.8–1.3)	0.233	0.809
Education (%)						0.228	0.438
No formal schooling	0 (0)	1 (0.0)	0 (0.0)	0 (0)	1 (0)	‐	‐
Less than secondary school	2 (0.2)	5 (0.2)	3 (0.1)	3 (0.2)	6 (0.2)	‐	‐
Secondary school graduate	89 (8.0)	176 (6.7)	143 (6.6)	96 (6.4)	220 (7.4)	‐	‐
Some university	313 (28.2)	987 (37.8)	838 (38.7)	649 (42.93)	1136 (38.4)	‐	‐
University graduate	430 (38.7)	929 (35.6)	753 (34.7)	504 (33.3)	1040 (35.2)	‐	‐
Masters	161 (14.5)	361 (13.8)	300 (13.8)	177 (11.7)	388 (13.1)	‐	‐
Advance graduate degree^c^	115 (10.4)	151 (5.8)	130 (6.0)	84 (5.6)	165 (5.6)	‐	‐
Illnesses							
ARI, total	990	1903	1391	1466	5750	‐	‐
ARI per person, median (IQR)	1 (0–1)	1 (0–1)	0 (0–1)	1 (0–2)	1 (0–3)	‐	‐
Duration of ARI (days)	8 (7–15)	8 (7–14)	8 (7–15)	9 (7–17)	15 (7–29)	‐	‐
Influenza	17	13	57	60	147	‐	‐
Duration of influenza (days)	7.5 (7–10)	7 (6–10)	8 (6–11)	7 (6–10)	7 (6–10)	‐	‐
Hours present at hospital during ARI (i.e., no work missed)	306	608	381	NA	‐	‐	‐
Absent from work because of ARI (hours)	2539	5644	4317	NA	‐	‐	‐
Person‐time (years)	348	805	604	848	2605	‐	‐
ARI rate (per 100 py)	288	238	234	173	218	‐	‐
Influenza ARI rate (per 100 py)	16.0	3.5	13.7	7.2	10.0	‐	‐

Abbreviations: ARI, acute respiratory illnesses; IQR, interquartile range; NA, not available.

^a^ *P*‐values were obtained from univariate Poisson regression with the incidence of ARI (acute respiratory illness) or influenza as the response variable.

^b^ For year 2016 and 2017, there were five and six HCP, respectively, that had blank data entry for race variable; therefore, their races were treated as missing.

^c^ Professional degrees beyond a Master's degree (e.g., Ph.D.).

## 3 RESULTS

### 3.1 Demographics characteristics

During 2016–2019, we identified 5131 eligible HCP and enrolled 3150: 677 participated for one study‐year, 761 for two study‐years, 1024 for three study‐years, and 688 for four study‐years. Of these, 2968 (93%) contributed person‐time (total of 2605.2 person‐years [py]) and became our analytic sample. Among the 2968 HCP, 657 participated in only 1 year, 783 participated in 2 years, 923 participated in 3 years, and 605 participated in all 4 years of the study period. Their median age was 41 years (interquartile range [IQR] 32–51), and the majority (2138, 72%) was female. Most (89%) were mixed race/ethnicity (*n* = 2640), 5.2% Quechua (*n* = 153), 2.1% Afro‐Peruvian (*n* = 58), and 1.3% White or European (*n* = 38). More than a third (38.5%, 1143) were married and lived in a dwelling with a median of one person per room (IQR 0.8–1.3). More than 1 in 4 HCP (894, 30.1%) drank >2 alcoholic beverages on any given day, and 376 (13%) had asthma, 284 (9.6%) hypertension, 78 (2.6%) heart conditions, 71 (2.4%) neuromuscular disorders, 66 (2.2%) immunosuppression, and 54 (1.8%) kidney disorders (Table 1 and Table A1).

### 3.2 Vaccination and infection prevention practices

The 2968 HCP were physicians (*n* = 426, 15%), nurses (967, 33%), or other support personnel (1575, 53%). The HCP had an average of 14 years of experience working with patients (range: 1–46 years). The HCP reported washing their hands often (80%, 95% CI 60–98%) during situations they judged required handwashing. Fifty‐eight percent of HCP (*n* = 1710) performed aerosolizing procedures; 6% (187) wore well‐fitting masks and goggles during these procedures. Although 1412 (49%) were vaccinated against influenza at least once during 2016–2019, only 36 (6%) of the 605 HCP who participated in all 4 years were vaccinated every study year (Table 1 and Table A1).

### 3.3 ARI and laboratory‐confirmed influenza and other respiratory viruses among HCP

During the 2016–2019 influenza seasons, the 2968 HCP had a total of 5750 ARI and provided 3054 respiratory swabs; 814 (27%) of these 2968 had ARI but did not provide timely swabs for testing. These HCP had a total of 2426 ARIs (586 in Year 1, 794 in Year 2, 567 in Year 3, and 479 in Year 4). Overall, the HCP had a median of one ARI (IQR 0–3) and 15 ARI illness‐days (IQR 7–29 days) during the study. For years with available data about presenteeism and absenteeism, (i.e., 2016–2018), the HCP with ARI were present at work while ill for a cumulative 1436 hours and absent because of ARI for a cumulative 12,500 hours. One hundred and forty‐three HCP had one rt‐PCR‐confirmed influenza ARI event and two HCP had two rt‐PCR‐confirmed influenza events (total of 147 influenza ARI during 2016–2019); 37 of these 147 (25%) were vaccinated. Influenza illnesses lasted a median of 8 days (IQR 6–11 days). The HCP with laboratory‐confirmed influenza were present at work while ill for a cumulative 1187 hours. A convenience sample of 699 ARIs that tested negative for influenza were reflex‐tested through FilmArray: 185 (26%) were positive for rhinoviruses or enteroviruses, 76 (11%) for seasonal coronaviruses (i.e., 40 [6%] for OC43, 17 [2%] for NL63, 12 [2%] for HKU1, and 7 [1%] for 229E), 11 (2%) for respiratory syncytial virus, and 9 (1%) for metapneumovirus (Table 1 and Table A1); none were positive for the three bacterial pathogens we tested for: *Bordetella pertussis*, *Chlamydophila pneumoniae*, and *Mycoplasma pneumoniae*.

Symptoms reported by the 147 rt‐PCR‐confirmed influenza cases were rhinorrhea (94%), cough (82%), myalgia (80%), sore throat (71%), headache (70%), fever (59%), chills (47%), fatigue (35%), difficulty concentrating (31%), shortness of breath (24%), earache (23%), wheezing (16%), nausea (10%), and diarrhea (6%). Sixty (41%) HCP sought care for their rt‐PCR confirmed influenza illnesses: 70% from outpatient clinics, 13% from emergency departments, and 17% at other locations. None required hospitalization. One‐hundred and four (71%) took medications, including antipyretics (46%), antibiotics (37%), antihistamines (33%), antitussives (6%), decongestants (3%); 1 participant (0.7%) took antivirals during their rt‐PCR confirmed influenza illnesses.

Throughout 2016–2019 influenza seasons, the incidence rate of ARI was 218/100py, and the age, sex, and occupation‐weighted influenza‐associated ARI was 10.0/100py (Table 1). The risk of influenza ARI was highest in 2018 (13.7/100py), when influenza A (H1N1) comprised 84% of influenza detections and lowest in 2017 (3.5/100py) when influenza A (H3N2) comprised 69% of detections (Table 1 and Table A1). In the analysis of pre‐ and post‐season sera, 19.1% (95%CI 17.2–21.1%) of asymptomatic and unvaccinated HCP developed influenza antibodies at the end the influenza epidemics (i.e., 147 [22.4%] of 657 in 2016, 18 [14.8%] of 122 in 2017, 28 [18.7%] of 150 in 2018, and 103 [16.5%] of 623 in 2019).

### 3.4 Factors associated with ARI and influenza

Although the risk of ARI in multivariable modeling was significantly higher among female HCP (aRR 1.1, 95%CI: [1.05, 1.25]) compared with male HCP, the risk of ARI in nurses, technicians, therapists, and physicians was similar to that of medical assistant support personnel (i.e., the referent group). The risk of ARI was greater among the HCP with asthma (aRR 1.3, 95%CI: [1.23, 1.47]), hospitalization in the prior year (aRR 1.2, 95%CI: [1.11, 1.35]), and those who directly cared for pediatric patients (aRR 1.1, 95%CI: [1.03, 1.18]). The HCP who wore a well‐fitting mask (i.e., fit‐tested or given a respirator that correctly fit) and goggles while performing aerosolizing procedures had a significantly lower risk of ARI (aRR 0.8, 95%CI: [0.68, 0.94]) compared with those who did not (Table 2). Although using well‐fitting mask and goggles during aerosolizing procedures was not associated with laboratory‐confirmed influenza risk (Table A1), the HCP who cared for pregnant women had increased risk of laboratory‐confirmed influenza (aRR 1.7, 95%CI: [1.09, 2.56]) (Table 3).

**TABLE 2:** Summary of Poisson regression model and relative rates for acute respiratory illness among healthcare personnel—Vacunas de influenza Peru, 2016–2019.

Variables	Adjusted incidence rate (per 100 py)	*P*‐value^b^	‐	Relative rate (95% CI)^a^
Age (year)	286 (269, 305)^c^	0.10	(Per 1 unit increase)	1.00 (1.00, 1.01)
Sex				
Female	264 (241, 290)	0.00	(Female vs. male)	1.14 (1.05, 1.25)
Male	231 (207, 257)	‐	‐	‐
Occupation				
Nurses, technicians, therapists	241 (217, 267)	0.42	(Nurses vs. physicians)	0.93 (0.84, 1.04)
Physicians	258 (230, 289)	‐	(Physicians vs. MASP)	1.07 (0.96, 1.18)
Medical assistants support personnel	242 (219, 267)	‐	(MASP vs. purses)	1.00 (0.93, 1.08)
Year				
2016	314 (284, 349)	0.00	(2016 vs. 2017)	1.23 (1.15, 1.32)
2017	255 (232, 280)	‐	(2017 vs. 2018)	1.01 (0.95, 1.07)
2018	252 (228, 278)	‐	(2018 vs. 2019)	1.37 (1.28, 1.45)
2019	184 (167, 204)	‐	‐	‐
Well‐being self‐assessment score	257 (253, 261)^c^	0.00	(Per 1 unit increase)	0.99 (0.98, 0.99)
Asthma				
Yes	286 (256, 319)	0.00	(Yes vs. No)	1.33 (1.23, 1.47)
No	213 (195, 234)	‐	‐	‐
Wore well‐fitting mask^d^ and goggles				
Yes	221 (189, 259)	0.00	(Yes vs. No)	0.81 (0.68, 0.94)
No	275 (258, 294)	‐	‐	‐
Cared for patients aged 13–19 years				
Yes	259 (235, 286)	0.01	(Yes vs. No)	1.09 (1.03, 1.18)
No	235 (213, 259)	‐	‐	‐
Hospitalization in prior year				
Yes	273 (242, 308)	0.00	(Yes vs. No)	1.22 (1.11, 1.35)
No	223 (205, 243)	‐	‐	‐

Abbreviation: CI, confidence interval.

^a^ Relative rate for each variable is adjusted for all other variables in the selected model.

^b^ *P*‐values were obtained from selected Poisson regression.

^c^ The baseline adjusted incidence rates for continuous variables (range for age: 19–72 years; range for well‐being self‐assessment score: 8–100).

^d^ Participants were deemed to have used well‐fitting mask if they confirmed they fit‐tested or were given a respirator that correctly fit their face or given an opportunity to pick a respirator that correctly fit their face.

**TABLE 3:** Relative rates of influenza illness among healthcare personnel—Vacunas de influenza Peru, 2016–2019.

Variables	Adjusted incidence rate (per 100py)	*P*‐value^b^		Relative rate^a^ (CI)
Age (years)	1.6 (1.5, 1.8)^c^	0.69	(Per 1 unit increase)	1.00 (0.98, 1.01)
Sex				
Female	7.1 (5.4, 9.3)	0.36	(Female vs. male)	1.26 (0.75, 2.14)
Male	5.6 (3.6, 8.7)	‐	‐	‐
Occupation				
Nurses, technicians, therapists	5.9 (4.0, 8.7)	0.60	(Nurses vs. physicians)	0.77 (0.42, 1.41)
Physicians	7.7 (4.8, 12.4)	‐	(Physicians vs. MASP)	1.41 (0.75, 2.64)
Medical assistants, support personnel	5.5 (3.7, 8.0)	‐	(MASP vs. nurses)	0.93 (0.60, 2.01)
Year				
2016	8.0 (4.6, 13.8)	0.00	(2016 vs. 2017)	3.95 (1.85, 8.44)
2017	2.0 (1.1, 3.6)	‐	(2017 vs. 2018)	0.17 (0.09, 0.32)
2018	11.7 (8.7, 15.8)	‐	(2018 vs. 2019)	1.41 (0.96, 2.07)
2019	8.3 (6.1, 11.3)	‐	‐	‐
Cared for pregnant patients				
Yes	8.1 (5.6, 11.8)	0.04	(Yes vs. No)	1.67 (1.09, 2.56)
No	4.9 (3.7, 6.5)	‐	‐	‐

Abbreviation: CI, confidence interval.

^a^ Relative rate for each variable is adjusted for all other variables in the selected model.

^b^ *P*‐values were obtained from selected Poisson regression.

^c^ The baseline adjusted incidence rates for continuous variable (range for age: 19–72 years) among HCP with rt‐PCR confirmed respiratory swabs.

## 4 DISCUSSION

In Peru, during 2016–2019, HCP infrequently got vaccinated against influenza, frequently developed ARI during influenza epidemics, and one in 20 tested positive for influenza; although more than one in three HCP took antibiotics for their influenza illness, only one participant took antivirals. The incidence of influenza among HCP seemed higher than that of community‐dwelling persons in Peru aged 18–49 years (6.4/100 py),^12^ among adults in other countries (9%),^19^ and pregnant women in Peru (88.7/10000 pregnant woman‐months).^20^ Although subtle changes in the way investigators estimate incidence and year‐to‐year variability in influenza circulation make it difficult to compare our findings with those of other studies,^21^ our findings suggest that HCP in Peru were at high risk of developing influenza infections and illnesses.^11^ Predictably, the risk of influenza accrued during the four‐year study period such that by 2019, one in 20 participants had an influenza illness. Furthermore, for each season, one in five asymptomatic and unvaccinated HCP developed new influenza antibodies by the end of the influenza season, suggesting that these HCP probably had asymptomatic influenza infections or paucisymptomatic illnesses. Although it is unclear how frequently HCP infect others at work, especially if they are wearing personal protective equipment, HCP with paucisymptomatic influenza illnesses or asymptomatic influenza infection could theoretically infect others. Given that HCP worked at these hospitals for dozens of years, on average, one might imagine a proportional increase in the work‐lifetime cumulative risk of influenza contagion.

Although HCP had self‐limiting ARI and seldom required advanced care, they typically had more than a week of respiratory symptoms and substantial presenteeism, absenteeism, and care‐seeking. The HCP in our cohort were present at their hospitals while ill for more than a thousand cumulative hours during the study, when they were likely to shed viruses and expose colleagues and patients to contagion. The HCP with laboratory‐confirmed influenza developed symptoms typical of ARI. Nevertheless, one in four HCP had difficulty concentrating, and one in five had symptoms that could distract and compromise clinical care, such as fatigue and shortness of breath. Such findings suggest the value of revisiting stay‐at‐home‐while‐ill and paid time‐off policies to avoid HCP presenteeism and the risk of compromised clinical care and nosocomial contagion. Similarly, the HCP were absent from work for 12,500 hours during the study period, potentially limiting hospitals' capacity to provide care during these epidemics.

Three out of four HCP with laboratory‐confirmed influenza took medications during the study period and, although one in three used antibiotics, only one used an antiviral to treat their viral influenza illness. Although prescriptions are required for the purchase of antibiotics and antivirals in Peru, such regulations are not enforced, and HCP have ready access to these with or without a prescription through local pharmacies and colleagues. The use of antibiotics for ARI, which are typically caused by viruses, increases the risk of antimicrobial resistance and the cost of care without benefiting those who are ill.^22^ Instead of antibiotics, HCP might have only sought symptomatic care with over‐the‐counter medications or empiric antiviral treatment to shorten the duration of presumptive influenza illness. Empiric antiviral treatment is especially recommended, within hours of symptom onset, for persons at increased risk of influenza complications such as the one in four HCP with underlying chronic conditions, HCP aged ≥65 years, or HCP with progressive illness, (e.g., new onset shortness of breath) as antivirals can decrease the risk of influenza hospitalization and death.^23^ While the government of Peru recommended empiric antiviral treatment for influenza illnesses as early as 2009, it is unclear if these are frequently stocked in Lima pharmacies, routinely used for clinical care, or cost‐effective in middle‐income settings. In the future, health authorities could reexamine the value of early empiric antiviral treatment during influenza epidemics for persons in middle‐income countries at increased risk of influenza illness complications.

Despite annual campaigns that promoted free‐of‐charge influenza vaccination at study hospitals, less than half (49%) of the HCP were vaccinated against influenza at least once during the study period, and only one in 15 (6%) were vaccinated in all 4 years.^24^ The HCP in our study used influenza vaccines less often than those surveyed during the 2009–2010 pandemic (i.e., 77.2%).^6^ PAHO and other health authorities recommend that HCP use vaccines to protect themselves from influenza illnesses, decrease the risk of nosocomial infections, prevent absenteeism, protect healthcare system surge capacity during epidemics and pandemics, and increase the likelihood that HCP will recommend vaccines to other target groups.^1^

It is unclear why freely available influenza vaccines in Peru, administered at the workplace, were underutilized.^24^ Common reasons why HCP might not seek vaccination are uncertainty about their individual risk of influenza and the value of vaccines.^1^ Indeed, HCP might be more accustomed to judging the risk of illness and the benefit of interventions for individuals but less so for public health interventions, which benefits accrue in communities.^25^ Although this study provides evidence about the risk and disruption associated with influenza illness, further evaluations are needed to optimize strategies to immunize HCP against influenza.^26^ A recent prospective cohort study of HCP in Israel and Peru, which included some of the participants in our Peru VIP cohort, estimated that standard‐dose influenza vaccine effectiveness against illness was on average null during six study seasons.^26^ Additional studies are necessary to determine if cell‐based or other enhanced influenza vaccines might be more effective at preventing influenza in this population frequently exposed to influenza viruses.^27^

Non‐pharmaceutical interventions have been effective in reducing contagion with influenza and other respiratory virus contagions during the COVID‐19 pandemic.^28^ Our multivariable models demonstrate how those who wore well‐fitting mask and goggles during aerosolizing procedures had lower risk of ARI. Hospital authorities might therefore continue to facilitate a culture of infection control through environmental and social engineering that protects both HCP and their patients. Some non‐pharmaceutical interventions, such as frequent hand washing, and mask‐wearing might be cost‐effective in preventing contagion during influenza epidemics.

Our study had strengths and limitations. We followed HCP during multiple seasons, through active follow‐up twice a week, and used sensitive case definitions and molecular diagnostics to identify laboratory‐confirmed influenza illnesses. Such methods yielded similar estimates to those of other carefully operationalized cohorts.^20^ Nevertheless, we relied on enrolment surveys for information to describe HCP characteristics. Such an approach might increase the probability of misclassification of exposures such as handwashing frequency, which is notoriously sensitive to desirability bias, can change over time, and is better assessed through observation.^29^ Future evaluations could include subroutines where study staff observe and record instances of handwashing behavior and mask use, by type and during specific procedures, rather than relying on recall and self‐reported behavior. Nevertheless, such misclassification could be expected to obscure rather than identify spurious associations between HCP characteristics and the risk of ARI and influenza. Second, the size of our cohort, identification of ARI, effective respiratory swabbing, our ability to laboratory‐confirm influenza, and our confidence in year‐specific ARI and seroconversion rates varied during each season during 2016–2019. We have added confidence intervals to our rates to help readers better appreciate this variability. It is also probable that despite the use of a sensitive case‐definition, we failed to identify all ARI, especially among paucisymptomatic HCP. This would have underestimated ARI and influenza rates, presenteeism, and absenteeism, especially among those with very mild illnesses. Last, although we designed our cohort to optimize its internal validity and its results seem concordant with contemporaneous cohorts in the subregion,^12^, ^18^, ^20^ our findings should be generalized with caution.

## 5 CONCLUSION

HCP frequently had ARI during the influenza season, approximately one in 20 were rt‐PCR‐positive for influenza illness, and one in five developed influenza antibodies compatible with paucisymptomatic or asymptomatic infection. Illness was associated with approximately 1 week of symptoms, including those that could distract from clinical duties. HCP were present at the hospital while ill and missed work because of illness, potentially decreasing hospital surge capacity during influenza epidemics. Less than half of HCP used influenza vaccines at the beginning of influenza epidemics and only one used an antiviral to treat their influenza illness. Further evaluation of the reasons for such choices and the potential value of continuing medical education to strengthen infection control practice seems warranted.

## AUTHOR CONTRIBUTIONS

**Eduardo Azziz‐Baumgartner:** Conceptualization; investigation; methodology; writing—original draft; writing—review and editing. **Joan Neyra:** Conceptualization; data curation; investigation; writing—original draft; writing—review and editing. **Tat Yau:** Formal analysis; writing—review and editing. **Giselle Soto:** Conceptualization; data curation; methodology; validation. **Daniel Owusu:** Formal analysis; validation; writing—review and editing. **Chao Zhang:** Formal analysis; writing—original draft; writing—review and editing. **Candice Romero Romero:** Conceptualization; data curation; methodology; validation. **Young M Yoo:** Formal analysis; writing—review and editing. **Miriam Gonzales:** Investigation; writing—review and editing. **Yeny Tinoco:** Conceptualization; data curation; methodology; writing—review and editing. **Maria Silva:** Investigation; methodology; writing—review and editing. **Eduar Bravo:** Data curation; validation; writing—review and editing. **Nancy Rojas Serrano:** Writing—original draft; writing—review and editing. **Eduardo Matos:** Data curation; validation; writing—review and editing. **Victor Chavez‐Perez:** Data curation; validation; writing—review and editing. **Juan Carlos Castro:** Data curation; validation; writing—review and editing. **Maria Esther Castillo:** Data curation; validation; writing—review and editing. **Rachael Porter:** Conceptualization; data curation; validation; writing—review and editing. **César Munayco:** Validation; writing—review and editing. **Angel Rodriguez:** Investigation; writing—review and editing. **Min Levine:** Formal analysis; investigation; methodology; validation; writing—review and editing. **Michael Prouty:** Investigation; writing—review and editing. **Mark Thompson:** Conceptualization; investigation; methodology. **Carmen Sofia Arriola:** Conceptualization; investigation; methodology; validation; writing—review and editing.

## CONFLICT OF INTEREST STATEMENT

None of the authors report conflicts of interest.

### PEER REVIEW

The peer review history for this article is available at https://www.webofscience.com/api/gateway/wos/peer-review/10.1111/irv.13189.

## ETHICS APPROVAL AND PATIENT CONSENT

The VIP cohort protocol was reviewed and approved by NAMRU‐6, Abt Associates (i.e., the institution coordinating the study for the CDC), and each study hospital. Participants provided written informed consent to participate. CDC funded the study through a contract with Abt Associates and an inter‐agency agreement with the US Naval Medical Research Unit No. 6. CDC staff guided study design, protocol development, and de‐identified data analyses and interpretation; CDC staff also analyzed de‐identified data and drafted this manuscript. This study was reviewed and approved by the NAMRU‐6 IRB as the single IRB for this study.*

## DISCLAIMER

The findings and conclusions in this article are those of the authors and do not necessarily reflect the official policy or position of the Department of the Navy, Department of Defense, the Centers for Disease Control and Prevention, nor the United States Government.

## Data Availability

Data may be available upon a written request to the corresponding author.

## Footnotes

* See 45 C.F.R. part 46.114; 21 C.F.R. part 56.114.

## APPENDIX A

**TABLE A1:** Household characteristics, underlying conditions, vaccination, and infection prevention practices among healthcare personnel—Vacunas de Influenza Peru, 2016–2019.

Variable	Year of enrollment					*P*‐value^a^	
	2016	2017	2018	2019	2016–2019^b^	ARI	Influenza
Monthly household income ($) n (%)						0.873	0.232
≤750	35 (3.2)	30 (1.2)	27 (1.2)	20 (1.3)	37 (1.2)	‐	‐
751–3000	475 (42.7)	1252 (48.0)	1065 (49.2)	794 (52.5)	1441 (48.7)	‐	‐
3001–6000	244 (22.0)	545 (20.9)	448 (20.7)	296 (19.6)	605 (20.5)	‐	‐
6001–9000	80 (7.2)	167 (6.4)	139 (6.4)	77 (5.1)	178 (6.0)	‐	‐
9001–12,000	56 (5.0)	90 (3.4)	81 (3.7)	49 (3.2)	103 (3.5)	‐	‐
12,001–15,000	33 (3.0)	57 (2.2)	45 (2.1)	29 (1.9)	62 (2.1)	‐	‐
15,001–18,000	18 (1.6)	40 (1.5)	27 (1.3)	17 (1.1)	45 (1.5)	‐	‐
18,001–21,000	11 (1.0)	25 (1.0)	21 (1.0)	20 (1.3)	28 (1.0)	‐	‐
21,001–24,000	9 (0.8)	15 (0.6)	12 (0.6)	9 (0.6)	15 (0.5)	‐	‐
>24000	5 (0.4)	5 (0.2)	4 (0.2)	2 (0.1)	5 (0.2)	‐	‐
Refused	145 (13.1)	384 (14.7)	298 (13.8)	200 (13.2)	438 (14.8)	‐	‐
Health upon enrolment n (%)							
Socioeconomic status self‐assessment (range 1–9)	5 (5–6)	5 (5–6)	5 (5–6)	5 (5–6)	5 (5–6)	0.808	0.441
Well‐being self‐assessment, median (IQR)	80 (70–90)	80 (70–90)	80 (70–90)	80 (70–90)	80 (70–90)	**0.000**	0.679
Days per week of physical exercise, which results in sweating, median (IQR)	0 (0–3)	0 (0–3)	0 (0–3)	0 (0–3)	0 (0–3)	**0.041**	0.746
Binge drinking alcohol (i.e., >2 drinks/day for males, >1 for females)	321 (28.8)	789 (30.2)	658 (30.4)	471 (31.19)	894 (30.1)	0.094	0.714
Smoker (ever)	98 (8.8)	221 (8.5)	194 (9.0)	121 (8.0)	256 (8.7)	0.631	0.365
Cigarettes per day among current smokers	3.5 (2.5–5.5)	3 (2–5)	3 (2–5)	3 (2–6)	3.5 (2–5)	0.347	0.277
Preexisting conditions n (%)							
Underweight	4 (0.4)	10 (0.4)	10 (0.5)	7 (0.5)	12 (0.4)	‐	‐
Obesity	203 (18.3)	459 (17.6)	397 (18.3)	269 (17.8)	526 (17.8)	0.529	0.556
Asthma	137 (12.3)	338 (12.9)	271 (12.5)	181 (12.0)	376 (12.7)	**0.000**	0.350
Hypertension	123 (11.0)	257 (9.8)	204 (9.4)	127 (8.4)	284 (9.6)	**0.007**	0.149
Other medical condition	149 (13.4)	306 (11.7)	256 (11.8)	174 (11.5)	346 (11.7)	**0.008**	0.641
Hospitalization in prior to enrolment	97 (8.7)	244 (9.3)	190 (8.8)	154 (10.2)	275 (9.3)	**0.001**	0.227
Other chronic lung conditions	4 (0.4)	12 (0.5)	8 (0.4)	9 (0.6)	14 (0.5)	‐	‐
Diabetes	39 (3.5)	82 (3.1)	70 (3.2)	53 (3.5)	95 (3.2)	0.073	0.490
Heart conditions	26 (2.3)	70 (2.7)	59 (2.7)	41 (2.7)	78 (2.6)	**0.016**	0.330
Neuromuscular disorders	32 (2.9)	63 (2.4)	47 (2.2)	36 (2.4)	71 (2.4)	**0.023**	0.081
Immunosuppression	31 (2.8)	60 (2.3)	50 (2.3)	38 (2.5)	66 (2.2)	0.117	0.672
Cancer	22 (2.0)	43 (1.6)	39 (1.8)	27 (1.8)	52 (1.8)	0.138	0.812
Kidney disorders	28 (2.5)	47 (1.8)	35 (1.6)	23 (1.5)	54 (1.8)	0.121	0.226
Occupation n (%)						0.323	0.703
Physicians	225 (20.2)	388 (14.8)	299 (13.8)	143 (9.5)	426 (14.4)	‐	‐
Nurses, technicians, & therapists	393 (35.2)	892 (34.1)	743 (34.3)	500 (33.0)	967 (32.6)	‐	‐
Medical assistants & support personnel	498 (44.6)	1336 (51.1)	1125 (51.9)	870 (57.5)	1575 (53.1)	‐	‐
Years of experience	13 (6–24)	12 (5–21)	12 (5–22)	12 (6–22)	12 (5–22)	**0.002**	0.929
Hours with patient per week	30 (20–36)	30 (20–36)	30 (20–36)	30 (20–36)	30 (20–36)	0.231	0.858
Number (%) of HCP by their patients' age group or pregnancy status n (%)							
Infants (<1 year)	268 (24.0)	926 (35.4)	666 (30.7)	350 (23.1)	999 (33.7)	**0.001**	0.459
Children aged 1–12 years	287 (25.7)	1031 (39.4)	734 (33.9)	377 (24.9)	1117 (37.6)	**0.019**	0.190
Children aged 13–19 years	376 (33.7)	1085 (41.5)	842 (38.9)	508 (33.6)	1196 (40.3)	**0.003**	0.230
Adults aged 20–64	899 (80.6)	1800 (68.8)	1611 (74.3)	1275 (84.3)	2088 (70.4)	0.238	0.801
Adults aged >65	528 (47.3)	1026 (39.2)	933 (43.0)	738 (48.8)	1197 (40.3)	0.208	0.843
Pregnant women	285 (25.5)	620 (23.7)	539 (24.9)	427 (28.2)	707 (23.8)	0.100	**0.031**
Procedures n (%)							
Swabs patients	81 (7.3)	218 (8.3)	174 (8.0)	116 (7.7)	239 (8.1)	0.697	0.185
Obtains sputum	268 (24.0)	580 (22.2)	494 (22.8)	359 (23.7)	653 (22.0)	0.624	0.607
Nebulizes medications	309 (27.7)	723 (27.6)	595 (27.5)	409 (27.0)	794 (26.7)	0.344	0.868
Applies O_2_ by nasal cannula	404 (36.2)	966 (36.9)	804 (37.1)	577 (38.1)	1064 (35.9)	0.663	0.894
Applies oxygen mask	473 (42.4)	1026 (39.2)	850 (39.2)	610 (40.3)	1141 (38.4)	0.995	0.078
Intubates tracheas	150 (13.4)	222 (8.5)	178 (8.2)	107 (7.1)	245 (8.3)	0.526	0.303
Inserts nasogastric tubes	283 (25.4)	595 (22.7)	478 (22.1)	313 (20.1)	644 (21.7)	0.359	0.463
Manually ventilates	214 (19.2)	457 (17.5)	341 (15.7)	189 (12.5)	494 (16.6)	0.769	0.491
Mechanically ventilates	166 (14.9)	328 (12.5)	252 (11.6)	156 (10.3)	349 (11.8)	0.992	0.338
Applies suction	384 (34.4)	666 (25.5)	551 (25.4)	370 (24.4)	722 (24.3)	0.687	0.105
Provides chest physiotherapy	140 (12.5)	313 (12.0)	243 (11.2)	147 (9.7)	338 (11.4)	0.723	0.176
Does bedside bronchoscopy	30 (2.7)	38 (1.5)	35 (1.6)	23 (1.5)	42 (1.4)	0.820	0.057
Self‐Reported infection control n (%)							
Gloves	678 (60.7)	1420 (54.3)	1186 (54.7)	823 (54.4)	1582 (53.3)	0.582	0.299
Gown	505 (45.2)	1045 (40.0)	882 (40.7)	643 (42.5)	1172 (39.5)	0.827	0.437
Cloth masks	217 (19.4)	528 (20.2)	418 (19.3)	238 (15.7)	581 (19.6)	0.986	**0.028**
Surgical masks	135 (12.1)	352 (13.5)	288 (13.3)	169 (11.2)	394 (13.3)	0.773	0.353
Respirator	544 (48.7)	1078 (41.2)	927 (42.8)	692 (45.7)	1199 (40.4)	0.066	0.188
Goggles	135 (12.1)	225 (8.6)	188 (8.7)	121 (8.0)	258 (8.7)	0.051	0.630
Face shield	38 (3.4)	75 (2.9)	59 (2.7)	31 (2.0)	88 (3.0)	0.246	0.182
Well‐fitting mask^c^	443 (40.2)	863 (33.3)	753 (35.0)	567 (37.8)	972 (33.0)	**0.011**	0.073
Fit testing	165 (15.3)	297 (11.7)	267 (12.7)	205 (14.1)	345 (12.0)	0.125	0.176
Any well fitted mask^c^ and goggles	100 (9.0)	165 (6.3)	140 (6.5)	93 (6.2)	187 (6.3)	**0.012**	0.452
Hand hygiene training	914 (82.8)	2160 (83.5)	1789 (83.1)	1309 (86.8)	2438 (83.1)	**0.013**	0.606
Number of times handwashing per 8–10‐h shift	15 (8–20)	15 (8–20)	15 (8–20)	15 (8–20)	15 (8–20)	0.119	0.409
Percent of situations that require handwashing % (IQR)	80 (60–95)	80 (60–99)	80 (60–99)	80 (60–95)	80 (60–98)	0.256	0.346
Influenza vaccine	428 (39)	656 (28.8)	719 (33.6)	372 (24.6)	1412 (49.2)	0.231	0.174

Abbreviations: ARI, acute respiratory illnesses; HCP, healthcare personnel; IQR, interquartile range.

^a^ *P*‐values were obtained from univariate Poisson regression with the incidence of ARI or influenza as the response variable.

^b^ This column contains results from HCP that participated in all 4 years during 2016–2019.

^c^ Participants were deemed to have used well‐fitting mask if they confirmed they fit‐tested or were given a respirator that correctly fit their face or given an opportunity to pick a respirator that correctly fit their face.
